# Rate-limiting steps in transcription dictate sensitivity to variability in cellular components

**DOI:** 10.1038/s41598-017-11257-2

**Published:** 2017-09-06

**Authors:** Jarno Mäkelä, Vinodh Kandavalli, Andre S. Ribeiro

**Affiliations:** 1 Laboratory of Biosystem Dynamics, BioMediTech Institute and Faculty of Biomedical Sciences and Engineering, Tampere University of Technology, 33101 Tampere, Finland; 2 Multi-scaled biodata analysis and modelling Research Community, Tampere University of Technology, 33101 Tampere, Finland; 3 CA3 CTS/UNINOVA. Faculdade de Ciências e Tecnologia, Universidade Nova de Lisboa, Quinta da Torre, 2829-516 Caparica, Portugal; 4 Present Address: Department of Biochemistry, University of Oxford, South Parks Road, Oxford, OX1 3QU UK

## Abstract

Cell-to-cell variability in cellular components generates cell-to-cell diversity in RNA and protein production dynamics. As these components are inherited, this should also cause lineage-to-lineage variability in these dynamics. We conjectured that these effects on transcription are promoter initiation kinetics dependent. To test this, first we used stochastic models to predict that variability in the numbers of molecules involved in upstream processes, such as the intake of inducers from the environment, acts only as a transient source of variability in RNA production numbers, while variability in the numbers of a molecular species controlling transcription of an active promoter acts as a constant source. Next, from single-cell, single-RNA level time-lapse microscopy of independent lineages of *Escherichia coli* cells, we demonstrate the existence of lineage-to-lineage variability in gene activation times and mean RNA production rates, and that these variabilities differ between promoters and inducers used. Finally, we provide evidence that this can be explained by differences in the kinetics of the rate-limiting steps in transcription between promoters and induction schemes. We conclude that cell-to-cell and consequent lineage-to-lineage variability in RNA and protein numbers are both promoter sequence-dependent and subject to regulation.

## Introduction

Single-cell measurements have shown that, even in monoclonal bacterial populations, cells differ widely in component numbers^1–6^. Most cell-to-cell variability in, e.g. RNA and protein numbers, in the regime of low molecule numbers, can be explained by the stochastic nature of biochemical reactions. Meanwhile, in the high molecule numbers regime, most variability is due to cell-to-cell variability in the numbers of molecules involved in gene expression^1^.

Fluctuations in molecular species numbers in a cell propagate through direct and indirect interactions between species^7, 8^. Also, noise from cellular processes such as DNA replication, and partitioning of molecules in cell division, also contribute significantly^9, 10^. Importantly, these fluctuations have non-negligible timescales, often longer than cells’ lifetime^1, 11, 12^, causing differences between sister cells to propagate to the timescale of cell lineages^13–15^.

Molecule number fluctuations likely affect most cellular processes. One process susceptible to these fluctuations is gene expression, as it depends on molecular species existing in small numbers (e.g. transcription factors) as well as on a cell’s abundance of polymerases, ribosomes, and σ factors^3, 14–19^.

At the single gene level, fluctuations in specific regulatory or uptake molecule numbers generate noise in the rates and timing of gene expression^4, 5, 13^. For example, gene expression activation rates by external inducers depend on the number of uptake membrane proteins^5^. As these differ in number between cells, so will intake times. Meanwhile, active transcription initiation rates (i.e. the main regulator of RNA production kinetics) differ due to, e.g., differences in the number of available RNA polymerases. It is expected that the effects of these noise sources in transcription will differ with the stage of gene expression affected.

Relevantly, the cell-to-cell variability in the kinetics of a chemical process depends not only on the variability in the numbers of the molecules involved, but also on the complexity of the process. For example, in a multi-step process such as transcription^6, 20–23^, the degree to which the cell-to-cell variability in RNA polymerase numbers (or another molecule involved in the process) affects the RNA numbers’ cell-to-cell variability, depends on the kinetics of all steps of the process. In particular, it is expected that only the duration of the first step (closed complex formation) will depend on the RNA polymerase numbers. As such, the larger the fraction of time in transcription initiation taken by the closed complex formation, the higher will be the effects of cell-to-cell variability in RNA polymerase numbers on the variability in RNA production kinetics. For example, if the closed complex formation takes only a small fraction of the overall duration of the process, even large deviations in its kinetics due to high variability in the numbers of the molecules involved (RNA polymerase, transcription factors, etc.) will not to cause major variability in the overall RNA production kinetics.

Thus, we hypothesize that promoters that differ in their sequence-dependent rate-limiting steps kinetics^21, 23–26^, will differ in their susceptibility to variability in molecule numbers. In addition, as the kinetics of the rate-limiting steps in transcription initiation are usually subject to regulation, e.g., by transcription factors^21, 27, 28^, we further hypothesize that the effects of cell-to-cell variability in molecule numbers can be tuned. Finally, as the time scale of fluctuations in molecule numbers and, thus cell-to-cell differences, can last longer than cell lifetimes and therefore propagate to cell lineages^1, 12, 13^, we expect that different promoters and different induction schemes will result in different lineage-to-lineage variability in RNA numbers.

To test these hypotheses, we combine stochastic modeling and time-lapse, single-cell, single-RNA level measurements of cell lineages to analyze the effects of variability in cellular components on transcription dynamics. Namely, we dissect the variability at each stage, from the external intake of inducers to the production of RNA molecules. For this, we first model transcription in cells accounting for the variability in numbers of the molecules involved in inducers intake and in transcription initiation rate constants, and study how these sources of variability contribute to the RNA variability over time. Next, to validate the model predictions, we measure differences in transcription dynamics between cell lineages. For this, we follow independent lineages for several generations under the microscope and measure RNA production in each lineage with single-cell, single-RNA sensitivity, to assess how the variability in gene activation rates following the introduction of inducers and in RNA production intervals in active promoters contribute to the lineage-to-lineage variability in RNA numbers over time. This variability is assessed and compared when inducing the same promoter, P_*lac/ara-1*_, with different inducers (IPTG and arabinose), and when inducing different promoters (P_*lac/ara-1*_ and P_*lac*_) with the same inducer (IPTG). Finally, we use different inducer concentrations to regulate the kinetics of the rate-limiting steps in transcription initiation, and study how this can be used to tune the propagation of noise in cellular component numbers into RNA numbers.

## Results

### Cell-to-cell variability in cellular components are expected to generate cell-to-cell variability in gene activation times and in active transcription kinetics

**Figure 1:**
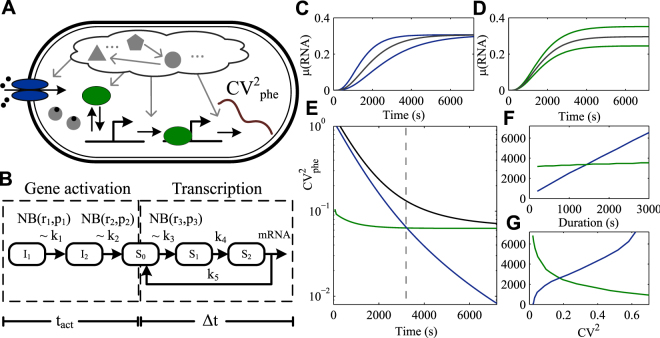
*In Silico* prediction of variability in RNA numbers from variability in molecule numbers in gene activation and in active transcription. (**A**) Schematic representation of unspecified intracellular processes affecting the kinetics of gene activation by external inducers and subsequent transcription that generate cell-to-cell variability in RNA numbers over time (CV^2^_phe_). (**B**) Gene activation (whose duration is represented by t_act_) is modeled as a stochastic 2-step process, while subsequent transcription events (whose overall duration is represented by Δt) are modeled as a stochastic 3-step process. The rates k_1_, k_2_, and k_3_ are proportional to the molecule numbers drawn from negative binomial distributions. (**C,D**) show the resulting median (gray) and the quartiles (blue in (**C**) and green in (**D**)) of the RNA numbers over time in cells differing in (**C**) uptake molecule numbers or (**D**) RNAp numbers. (**E**) CV^2^_phe_ resulting from differences in RNAp (green) or in uptake protein numbers (blue), and from differences in both (black). The dashed vertical line is the crossing time. From this figure, we find that cell-to-cell variability in uptake protein numbers contributes to RNA numbers diversity mostly at the early stages of a time series and then gradually dissipates, while noise in transcription is a constant source to RNA numbers diversity that dominates the latter stages of a time series. (**F**,**G**) show the effects on the crossing time of changing (**F**) the mean duration of the activation period (blue) and subsequent transcription events (green) and (**G**) the CV^2^ of uptake proteins (blue) and RNAp (green) numbers.

As in ref. 29, in each cell, we model gene activation and subsequent active transcription as stochastic multistep processes. Here, in addition, we impose that the rate of each step is dependent on the molecule number of specific molecular species (Fig. 1A and B). Specifically, the inducers’ intake kinetics from the environment differs with the number of uptake proteins^5^, while the rate of closed complex formation in transcription initiation differs with the numbers of free RNA polymerases (RNAp), as most active promoters are not saturated with holoenzymes^17, 30^. Thus, in this model, the cell-to-cell variability in uptake protein and RNAp numbers affect the variability in gene activation and subsequent transcription initiation rates, respectively, thus contributing to the cell-to-cell variability in RNA numbers.

Gene activation is the passage of a promoter from a non-producing to a producing state, following the appearance of an inducer in the media. It includes subsequent events such as diffusion of inducers in the extracellular and intracellular environments, crossing of the cell membranes, and finding and binding to a promoter or its repressor.^4, 31–33^ As these steps differ widely between genes, to model the dynamics of activation, we consider only the rate-limiting steps and model it as a two-step stochastic process as in refs 4, 29 (Supplementary Information):

1 $${{\rm{I}}}_{{\rm{1}}}\mathop{\longleftrightarrow }\limits^{{K}_{m}}{{\rm{I}}}_{{\rm{2}}}\mathop{\longrightarrow }\limits^{{k}_{v}}{{\rm{S}}}_{{\rm{0}}}$$

Here, I_1_ is a promoter in a non-producing state, I_2_ is an intermediate state, and S_0_ is a producing state, in which the promoter is available for transcription.

Active transcription in *E. coli* is a multi-step process, with the closed complex and open complex formation being, in most promoters, the most rate-limiting steps^21–23^. Transcription can thus be formulated as^22^:

2 $${\rm{RNAp}}+{\rm{Pro}}\mathop{\longleftrightarrow }\limits^{{{\rm{K}}}_{B}}{{\rm{RP}}}_{{\rm{c}}}\mathop{\longrightarrow }\limits^{{k}_{f}}{{\rm{RP}}}_{{\rm{o}}}\to \to \to {\rm{RNA}}$$

In (2), transcription initiates when an RNA polymerase holoenzyme (RNAp) binds to a promoter (Pro) and forms a closed complex (RP_c_). This step is reversible and thus, it takes several attempts, until one of them eventually successfully forms a stable open complex (RP_o_). Finally, the holoenzyme forms an elongation complex and synthesizes an RNA. The first-passage time distribution to produce an RNA is observationally equivalent to the distribution generated by a simplified version of the models in (1) and (2), shown in Fig. 1B (Supplementary Information)^26, 34^.

Each model cell contains a number of uptake proteins and RNAps that are drawn from negative binomial distributions of measured molecular species numbers^1^ (Supplementary Information). To attain RNA production dynamics in each cell, we used the finite state projection algorithm^35^, in which a finite set of linear ordinary differential equations is formulated for the truncated state space of the system to predict the time-varying probability distributions. From this, we obtain the RNA number distribution of a cell population over time.

To quantify and compare the effects of cell-to-cell variability in uptake protein and RNAp numbers, the variability in RNA numbers is described as^36^:

3 $${{\rm{CV}}}^{2}={{\rm{CV}}}_{{\rm{proc}}}^{2}+{{\rm{CV}}}_{{\rm{phe}}}^{2}$$

where

4 $${{\rm{CV}}}_{{\rm{proc}}}^{{\rm{2}}}=\frac{\overline{\langle {n}_{i}^{2}\rangle -{\langle {n}_{i}\rangle }^{2}}}{{\overline{(\langle {n}_{i}\rangle )}}^{2}},\,{{\rm{CV}}}_{{\rm{phe}}}^{{\rm{2}}}=\frac{\overline{{\langle {n}_{i}\rangle }^{2}}-{\overline{(\langle {n}_{i}\rangle )}}^{2}}{{\overline{(\langle {n}_{i}\rangle )}}^{2}}$$

Here, *n*_*i*_ is the number of RNAs in cells of a sub-population of cells with parameter values *i* (i.e. number of uptake proteins and RNAps); the bracket operator $\langle (\cdot )\rangle$ represents averaging over all cells with parameter values *i*; and the bar operator $\overline{(\cdot )}$ represents averaging over all values of *i*.

As the number of uptake proteins and RNAps are the features that can differ between cells, they are used here as the features that define the ‘phenotype’ of a cell. Overall variability in RNA numbers is generated by the process’ stochasticity (CV^2^_proc_) and by the differences in the cells’ propensities to produce RNAs (CV^2^_phe_), due to ‘phenotypic’ variability.

Note that the kinetics of gene activation and transcription do not differ between the cells. Effects of variability in these processes were studied in^26, 29^. Here, we focus on the effects of the ‘phenotypic’ variability (CV^2^_phe_) on the kinetics of activation and active transcription.

First, we studied the effects of cell-to-cell variability solely in uptake protein numbers. For that, the model cells do not differ in RNAp numbers. From Fig. 1C and E, this source of variability contributes to RNA numbers diversity mostly at the early stages of a time series. Once transcription becomes active in most cells, the uniform process of RNA degradation across the cell population causes its effects to gradually dissipate.

Next, we assumed no variability in numbers of uptake proteins and studied the effects of variability in RNAp numbers. Here, the initial stages of the time series exhibit much less cell-to-cell variability in RNA numbers (CV^2^_phe_) than the previous model. However, as transcription is activated throughout the cell population, its contribution to RNA numbers diversity becomes evident (Fig. 1D and E), being maximized when equilibrium is reached between RNA production and degradation.

Finally, we considered model cells where cell-to-cell diversity in both uptake protein and RNAp numbers are present. In these, in agreement with the above, the early stage of the time series is dominated by the variability in the gene activation process, while the latter stages are dominated by the variability in the transcription process (Fig. 1E). The moment when the latter overtakes the former is defined here as ‘crossing time’, and provides information about the duration of the influence from upstream processes. Importantly, the crossing time is often greater than a cell’s generation time, as shown in previous studies^4, 29^.

In addition, we quantified the dependence of the crossing time on the dynamics of activation and subsequent active transcription (Fig. 1F). We find that increasing the mean duration of gene activation increases the crossing time, as expected, while changing the active transcription initiation rate has only minimal effects. Also, the variability in RNAp and uptake protein numbers (measured by the CV^2^) affects the crossing time (Fig. 1G). Namely, increasing the CV^2^ of RNAp numbers decreases the crossing time, while increasing the CV^2^ of uptake protein numbers increases it.

### Variability in RNA numbers between lineages differs between promoters and their induction scheme

*E. coli* cells have been shown to behave more similarly in protein production kinetics when sharing a common ancestor due to inheritable epigenetic factors^13^. These factors are propagated to the progeny for several generations^1, 11, 12^, and thus cell lineages are expected to differ in these factors.

Given this, here we consider each independent lineage as a distinct phenotype, with a specific RNA production rate and inducer intake rate. To validate this assumption, we studied how individual cell lineages respond to transcription induction by measuring, over the course of several generations, the RNA production in each cell with single molecule sensitivity following the introduction of an inducer in the media.

**Figure 2:**
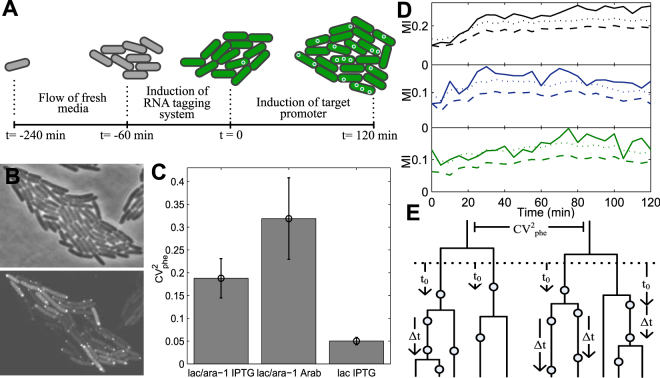
Variability in RNA production between lineages. (**A**) Cells are placed under the microscope at t = −240 min and continuously supplemented with fresh medium. At t = −60 min, the induction of the reporter system (MS2d-GFP) is initiated. At t = 0 min, with the cells already flooded with MS2d-GFP proteins for accurate RNA detection, the induction of the target RNA for MS2d-GFP is initiated. (**B**) Phase contrast image of an induced lineage and corresponding fluorescence image with tagged RNA molecules. (**C**) CV^2^_phe_ of the RNA numbers between lineages, 2 hours after induction. Shown are P_*lac/ara−1*_ induced with 1 mM IPTG (29 lineages) and with 1% Arabinose (14 lineages), and P_*lac*_ induced with 1 mM IPTG (60 lineages). Error bars are the standard errors determined by bootstrapping of the cells in the lineages (Fig. S3). Differences between conditions suggest that promoter sequence and transcription factors can regulate the CV^2^_phe_ in RNA production. (**D**) MI (solid line), sMI (dashed line) and 1-tailed 0.01 p-value (dotted line) between a cell’s lineage and the number of RNAs of each cell for P_*lac/ara-1*_ induced with 1 mM IPTG (black), P_*lac/ara-1*_ induced with 1% Arabinose (blue), and P_*lac*_ induced with 1 mM IPTG (green). In all conditions, the significant variability in the CV^2^_phe_ in RNA numbers arises during the induction process. (**E**) Illustration of RNA production events (circles) over time in individual cells of lineages. The waiting times for the first RNAs to appear in lineages (t_0_) and the subsequent time intervals between consecutive RNA production events (Δt) in single cells are shown. The dotted line depicts the start of induction of the target promoter.

We grew lineages from individual cells under the microscope, induced the reporter and target gene, and then measured the RNA production dynamics in each cell once the lineages reached a size of 40–50 cells (Fig. 2A). All data of each condition is from the same experiment to avoid differences between overnight cultures, gel properties, etc. We detected production of RNA molecules by MS2-GFP tagging method (Fig. 2B, Fig. S1, and Supplementary Information), which protects the target RNA from degradation for the duration of the measurements^37–39^. Parameters for the detection of the target RNA were kept the same between lineages to avoid biases in detection.

Measurements were conducted for differing inducers and promoters. Namely, we used a single copy P_*lac/ara-1*_ (inducible by arabinose and/or IPTG)^20^ and a single copy P_*lac*_ (inducible by IPTG)^37^. For P_*lac/ara-1*_ induced by 1 mM IPTG, P_*lac/ara-1*_ induced by 1% arabinose, and P_*lac*_ induced by 1 mM IPTG (in all cases for 2 hours), the cells exhibited, after 2 hours of induction, on average, 2.3, 0.4, and 3.0 RNAs, respectively, in agreement with previous *in vivo* measurements^1, 6^ (Supplementary Information, section ‘RNA numbers in cells’). It is noted that the strain used here was modified to contain a very high copy number of lac repressors (~3000 vs. ~20 in wild type)^20^ and to not code for lactose permease, which transports lactose into the cell. The first feature allows greatly increasing the fold change with induction when compared to the natural system. The second feature allows studying this system without the interference of feedback systems. In P_*lac/ara-1*_ promoter, the CRP/cAMP site has been replaced by the AraC binding sites of the P_*BAD*_ promoter to avoid pleiotropic effects and allow further activation of transcription^20^. Fig. S2 shows the topologies and sequences of the mentioned promoters.

To quantify the variability in RNA production dynamics between lineages, we obtained the CV^2^_phe_ of the lineages in each condition (Fig. 2C, Fig. S3). We find differences between all conditions, indicating that possibly both the intake (which differs with the inducer molecule) and the active transcription (which differs with the promoter sequence) processes affect the CV^2^_phe_ in RNA production of the lineages. Note that the CV^2^_phe_ is independent of the mean transcription initiation rate (Fig. S4).

Due to being limited to observe a finite number of cells and lineages, it is possible that these values differ solely due to random chance. To test this, we measured the mutual information (MI)^40^, which quantifies how much a variable informs about another, between the lineage and the RNA numbers of each cell. For comparison, we randomly permuted cells between lineages for 10^5^ times and calculated the average spurious MI (sMI), along with the 1-tailed p-value. The results are: P_*lac/ara-1*_ induced by IPTG (MI: 0.336, sMI: 0.258, p-value < 10^−5^); P_*lac/ara-1*_ induced by arabinose (MI: 0.138, sMI: 0.072, p-value < 10^−5^); P_*lac*_ induced by IPTG (MI: 0.185, sMI: 0.120, p-value < 10^−5^). Thus, in all conditions, the hypothesis of having obtained the measured variability in RNA numbers between lineages by random chance can be rejected. Also, to test whether the difference between the MI and sMI increases during the activation period of transcription following the addition of inducers, we obtained the MIs for each condition every 5 min for 2 hours (Fig. 2D). Initially, the MI and sMI are very similar but, as time advances, the MI increases rapidly, becoming significantly above the average sMI (and 1-tailed p-value of 0.01)(see also mean values for lineages in Fig. S5).

To test for the possibility that the inducer was not reaching all cells under observation, we calculated the correlation between the distance between a cell and the colony edge and its RNA numbers. In all conditions, we found only very weak, not statistically significant, spatial correlations (Table S1), meaning that the induction is approximately uniform in space. Also, we tested for reproducibility of the lineage variability from independent measurements by conducting three independent measurements for cells with P_*lac/ara-1*_ induced by IPTG. We observed no statistically significant differences between the measurements (Figs S6 and S7).

We conclude that, in all conditions, the variability between lineages in mean RNA numbers is significantly above chance. Further, it differs with both the promoter, which should affect the kinetics of active transcription, as well as with the inducer, which should affect the kinetics of both intake and active transcription.

### Contributions of gene activation and active transcription to lineage variability differ over time, with the former being transient and the latter being a constant source of variability

The observed lineage-to-lineage variability in RNA numbers can arise from gene activation, active transcription, or both. To assess the contribution of each process over time, we observed the waiting times for the first target RNA appearance (t_0_; which includes both t_act_ and Δt) in each cell present at the start of induction^29^, along with the time intervals between consecutive RNA production events in each cell (Δt)^29^ (Fig. 2E, Supplementary Information, Figs S1, S8 and S9). We extracted information from the same time-lapse experiment so as to minimize potential differences in environmental conditions. We also limited the observations to ~10 lineages per experiment to obtain sufficient time sampling. Results show that the CV^2^_phe_ in both gene activation times and transcription intervals between lineages differs between conditions (Table S2).

To validate that the time series data are representative of large populations of lineages, we compared the lineage-to-lineage variability in mean RNA numbers of the time series measurements to that of two independent measurements for the condition of P_*lac/ara-1*_ induced by IPTG. We observed no statistically significant differences (Figs S6 and S7).

**Figure 3:**
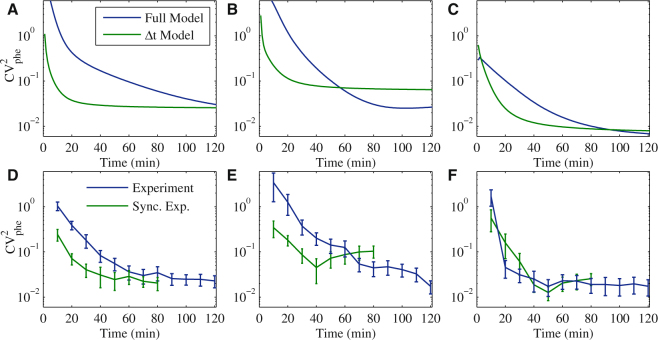
Lineages CV^2^_phe_ in RNA numbers over time. (**A**–**C**) CV^2^_phe_ in RNA numbers between lineages predicted for the full model (both t_0_ and Δt processes) and the Δt model over time. (**A**) model P_*lac/ara-1*_ induced with IPTG, (**B**) model P_*lac/ara-1*_ induced with arabinose and (**C**) model P_*lac*_ induced with IPTG. (**D**–**F**) CV^2^_phe_ in RNA numbers of measured and synchronized (sync) lineages. Branches of lineages without RNA production are discarded and, in the sync data, the last 40 minutes are not used due to the need for synchronization (**D**) P_*lac/ara-1*_ induced with IPTG (15 lineages), (**E**) P_*lac/ara-1*_ induced with arabinose (10 lineages), and (**F**) P_*lac*_ induced with IPTG (8 lineages). Error bars are standard errors determined by bootstrapping of the lineages. As predicted by the models, in all cases, the contributions from gene activation kinetics and from active transcription dynamics to the CV^2^_phe_ in RNA numbers differ over time, that the former has only a transient effect.

To estimate the contributions of each process to the observed lineage variability in RNA numbers over time, we fitted the measured t_0_ and Δt to the model of gene activation and transcription (Fig. 1B, Supplementary Information). We show results when assuming both activation (t_act_) and active transcription (Δt) (referred to as ‘full model’), and when assuming only active transcription (‘Δt model’) (Fig. 3A–C). In all conditions, the Δt model reaches a plateau, i.e. a constant CV^2^_phe_ faster than the full model. The height of this plateau is determined by the CV^2^_phe_ of Δt and is independent of the mean transcription initiation rate (Fig. S4, Table S2). The two conditions that differ the most in the time to reach the plateau are P_*lac/ara-1*_ induced by IPTG and P_*lac/ara-1*_ induced by arabinose. Further, under arabinose induction, the CV^2^_phe_ of the Δt model is initially higher, due to differences in the mean values of t_act_ and Δt. Over time, the two quantities will become similar (Fig. S10).

To compare with the model predictions, we calculated the empirical CV^2^_phe_ in RNA numbers over time. For this, we only considered branches of lineages where RNA productions occurred. The outcomes of the full models are expected to be representative of these measurements. Meanwhile, to obtain empirical values comparable with the Δt model, we synchronized the first production moment of RNA in each lineage to t = 0 and then disregarded that first production event. To avoid biases due the reduced number of cells in the later parts of the time series, we only considered the first 80 minutes of the synchronized time series.

The empirical lineages CV^2^_phe_ are shown for each condition, with and without synchronization (Fig. 3D–F). As predicted by the models, the CV^2^_phe_ of the synchronized lineages exhibits a plateau. Also, in P_*lac/ara-1*_, the CV^2^_phe_ of synchronized lineages reaches the plateau faster than the CV^2^_phe_ of non-synchronized lineages. Meanwhile, P_*lac*_ does not exhibit significant influence by the gene activation process on the lineages’ CV^2^_phe_. We expect that this is due to the higher leakiness of this promoter (Table S2). To test this notion, we studied the expected impact of leakiness on CV^2^_phe_ using a model that allows transcription in the absence of inducers. This leakiness was modelled as a Poisson process, and various rates of leakiness were tested. The results show that increasing leakiness decreases the lineages’ CV^2^_phe_ (Fig. S11).

Overall, these results confirm that the contributions from gene activation kinetics and from active transcription dynamics to the lineages CV^2^_phe_ in RNA numbers differ over time, and that the former has only a transient effect. Importantly, fluctuations in transcription kinetics act as a constant source of variability in RNA numbers between lineages that differs between conditions (i.e. between promoters and between induction mechanisms of the same promoter).

### Rate-limiting steps in transcription regulate the effects of cell-to-cell variability in cellular components on transcription kinetics variability

Why do the three conditions differ in variability between lineages (CV^2^_phe_) in the same strain? Promoter sequences have been shown to differ widely in the kinetics of the rate-limiting steps in transcription initiation^6, 21, 23, 24^. Also, depending on the molecular species whose numbers fluctuate, different stages of transcription are expected to be affected. For example, different transcription factors act at different stages and variability in their numbers affect mostly the variability in the kinetics of those stages alone.

Given this, we hypothesized that differences in the kinetics of the rate limiting steps as well as in which rate limiting steps are affected by differences in the numbers of transcription factors could be the source for the observed differences in CV^2^_phe_ between the conditions studied here. Let τ_cc_ represent the stages of transcription initiation whose kinetics depends on RNAp concentration, while τ_oc_ represents subsequent stages, which are independent of RNAp concentration^22, 26, 30, 41^. Given these definitions, we considered 4 different stochastic multi-step models of transcription (Fig. 1B) with the variability in molecule numbers affecting different rate-limiting steps: (1) variability in molecule numbers affecting only τ_cc_; (2) variability in molecule numbers affecting only τ_oc_; (3) variability in molecule numbers affecting both τ_cc_ and τ_oc_ equally; (4) variability in numbers of two molecular species (with different variabilities) affecting τ_cc_ and τ_oc_ independently. The extent of variability was set to be the same in all models (CV^2^ = 0.5) (except model 4, in which one molecular species has lower variability (CV^2^ = 0.1)) to reflect the empirical values reported in^7^. The overall RNA production rate was identical in all cases and does not affect the CV^2^_phe_ (Fig. S4). We studied the effects on CV^2^_phe_ of RNA numbers as a function of τ_cc_ relative to the overall duration of the transcription intervals, Δt.

**Figure 4:**
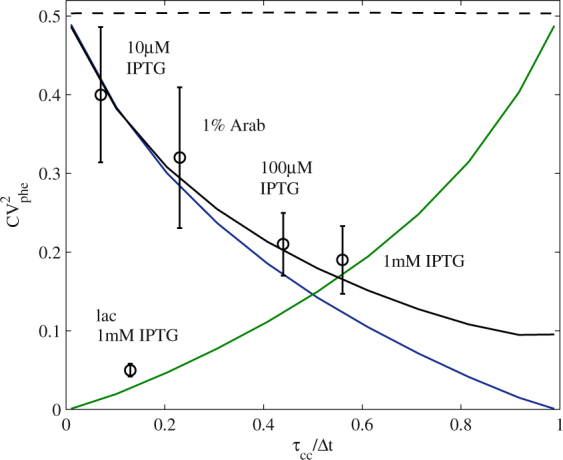
CV^2^_phe_ in RNA numbers as a function of τ_cc_/Δt as predicted by models and assessed by measurements. Lines are CV^2^_phe_ from stochastic models with variability in molecule numbers affecting τ_cc_ (green), τ_oc_ (blue), and both simultaneously (dashed line), as a function of τ_cc_/Δt. Also shown is a model with variability in numbers of two molecular species (with different variabilities) affecting τ_oc_ and τ_cc_ (black). Circles are the measured lineages CV^2^_phe_ as a function of τ_cc_/Δt. P_*lac/ara-1*_ induced with 10 µM IPTG (61 lineages), 100 µM IPTG (54 lineages), 1 mM IPTG (29 lineages), and 1% Arabinose (14 lineages). Also shown is P_*lac*_ induced with 1 mM IPTG (60 lineages). Error bars are standard errors determined by bootstrapping of the lineages. The same promoter subject to different induction levels influences its τ_cc_/∆t and will consequently differ in CV^2^_phe_ in a way that is predictable by our model of transcription. Also, different transcription factors result in different CV^2^_phe_.

The results (Fig. 4) show that CV^2^_phe_ varies with τ_cc_/Δt in models 1, 2, and 4, where the variability in molecule numbers affect τ_cc_ and τ_oc_ differently. In general, if variability in molecule numbers affects the longer lasting step, it results in higher CV^2^_phe_ in RNA numbers. This does not occur in model 3, because the variability in molecule numbers affects both rate-limiting steps equally. Overall, we conclude that it is possible to tune the effects of variability in molecular species affecting transcription by tuning the ratio between the durations of the rate-limiting steps in transcription initiation.

To provide empirical validation, we first measured the extent to which τ_cc_/Δt of P_*lac/ara-1*_ can be tuned by varying the IPTG concentration, as it has been shown that the kinetics of the rate-limiting steps can be regulated by inducers^21, 26^. The τ_cc_/Δt is obtained from τ-plots, as in ref. 26. For that, the inverse of the RNA production rate is plotted as a function of inverse of the relative RNAp concentration. Next, it is extrapolated for an “infinite” RNAp concentration, so as to obtain the relative value of τ_cc_ (Supplementary Information).

To alter RNAp concentrations in live cells, we used media with different concentrations of specific components, as described in ref. 26, and measured relative RpoC levels (i.e. the β’ subunit, which is the limiting factor in the assembly of the RNAp holoenzyme) in each condition by Western Blotting (Fig. S12, Supplementary Information). Importantly, it has been shown by qPCR and plate reader measurements that the inverse of the RNA production rate of P_lac/ara-1_ change linearly with the inverse of the total RNAp concentration within the range of media richness used in our measurements^26^.

Next, we measured by qPCR the fold-change in RNA production rates in each media compared to the control condition. Following this, τ_cc_/Δt was extracted from the τ-plot for each inducer condition (Fig. S13). Finally, for each condition, from microscopy measurements, we measured the lineages CV^2^_phe_ in RNA numbers after 2 hours of induction.

We show (Fig. 4) the experimental lineages CV^2^_ext_ for P_*lac/ara-1*_ for different IPTG concentrations (10 µM, 100 µM, and 1 mM) as a function of τ_cc_/Δt. Also shown are the results for P_*lac/ara-1*_ induced with 1% arabinose and P_*lac*_ induced with 1 mM IPTG. Notably, in P_*lac/ara-1*_, as τ_cc_/Δt increases, the lineages CV^2^_phe_ decreases. This behavior fits models 2 and 4, i.e., in this case the variability in molecule numbers influences mostly τ_oc_. Interestingly, in this regard, it is known that a bound lac repressor prevents open complex formation^27^. Similarly, AraC also affects the open complex formation^21^. This suggests that, in P_*lac/ara-1*_, the cell-to-cell variability in lac repressor and AraC numbers might be the sources of the lineages CV^2^_phe_ in RNA numbers.

P_*lac*_, on the other hand, exhibits much lower lineages CV^2^_phe_ (Fig. 4.) than those of P_*lac/ara-1*_, suggesting that its regulatory mechanisms and/or noise sources differ significantly from P_*lac/ara-1*_. Congruently, P_*lac*_ has fewer LacI binding sites than P_*lac/ara-1*_, and a CAP binding site, which facilitates closed complex formation^20, 21, 28, 42^ (Fig. S2). As such, P_*lac*_ is expected to have different contributions to transcriptional variability from the transcription factor.

We conclude that transcription factors can be used to indirectly control the propagation of variability from molecular species numbers, given their ability to tune the kinetics of the rate-limiting steps in transcription initiation. In addition, we expect that different promoters, differing in regulatory mechanism and/or noise sources^21–23^, will differ in responsiveness to molecular fluctuations.

## Discussion

It is well-known that the variability in cellular components, particularly in core regulators of gene expression, such as RNA polymerases, transcription factors, and ribosomes does not affect all genes uniformly (see e.g. ref. 19). i.e., the resulting degree of phenotypic variability is known to be genetic-background dependent. However, the causes for this dependency remain unclear. Here, we provided one likely molecular mechanism responsible for the gene-specific phenotypic variability. In particular, we considered that gene expression is a multi-step process, that genes differ in the duration of each step, and that each step is affected differently by changes in the numbers of the core regulators. Based on this, we hypothesized that genes have unique, tunable levels of susceptibility to the variability in cellular components and, particularly, to variability in the core regulators numbers.

Moreover, as the molecular components affecting transcription are inherited, cell-to-cell variability in RNA numbers should result in lineage-to-lineage variability in the same numbers. Consequently, transcription dynamics diversity between cells should result in transcription dynamics diversity between lineages whose degree, similarly to the cell-to-cell diversity, should differ between genes and with induction schemes.

In support of our hypothesis, we first showed that the lineage-to-lineage variability in mean RNA numbers differs between promoters and when inducing the same promoter with different inducers. Also, we showed that the former is due to differences in initiation kinetics between promoters, while the latter is due to different inducers leading to different active transcription initiation kinetics.

Aside from these sources of lineage-to-lineage variability, which have a constant effect over time, we further showed that the process of gene activation by an inducer acts as a transient source. Namely, we showed that differences in the kinetics of inducer intake during gene activation causes tangible differences in the lineage-to-lineage variability in mean RNA numbers, which gradually dissipate as all cells of the lineages become activated.

Next, to support our hypothesis that differences in the kinetics of the rate-limiting steps in transcription initiation allow genes to be affected differently by fluctuations in the numbers of molecular species involved in transcription, we showed that changing the inducer or its concentration, which changes the initiation kinetics of a promoter, changes the lineage-to-lineage variability. Also, different promoters subject to the same inducer exhibit different lineage-to-lineage variability. In particular, we showed that a source acting on the first step alone will have weak effects on promoters where this step is relatively fast, but will have strong effects on promoters where this step is the most rate-limiting one. These results indicate that the effects of variability in molecular species in the dynamics of transcription at the single cell level are subject to regulation and, in agreement with previous studies^7^, are evolvable at the single gene level.

In this regard, it is of interest to mention a recent study showed that selection on expression noise can have a stronger impact on sequence variation than mean expression level^43^. As such, it is of importance to identify which mechanisms cells can use to evolve noise levels of individual genes. The main contribution of our study, aside from the direct quantification and better understanding of the degree of diversity in RNA production kinetics between cells and lineages, is the identification of a mechanism, namely, the multi-step nature of transcription initiation, that allows the effects of extrinsic noise sources to be tunable by transcription factors and by the promoter sequence, which makes it both adaptable and evolvable.

Given the substantial fluctuations and cell-to-cell diversity known to exist in cellular components in *E. coli* cells^1^, we expect the promoter-level sensitivity to molecule number fluctuations to be a key factor for a reliable dynamics of small genetic circuits and cellular functioning in general. Also, given the evolvability and adaptability of the kinetics of the rate-limiting steps of transcription initiation, we expect that *E. coli* is constantly adjusting these features at the single gene level in order to reach optimal levels of functioning. Namely, we expect a global reduction of cell-to-cell and lineage-to-lineage diversity in RNA numbers when in stable environments, and, following a bet-hedging strategy, its rapid enhancement when exploring new environments.

In addition to this, since, in general, the intake kinetics of gene expression regulators is itself subject to regulation, it may be that this and the above regulatory mechanisms act and evolve in a combined fashion. Variability in molecules responsible for gene activation and activity can be generalized as a “signaling” level of regulation in individual cells that can affect the response and sensitivity of the transcriptional circuits to perturbation. Importantly, the differences in the initiation kinetics of the promoters of a small circuit, should allow these circuits to exhibit ‘circuit state-dependent’ or signal-specific reactions. For example, consider a genetic switch where the initiation kinetics of promoter 1 is mostly spent in closed complex formation, while in promoter 2 it is mostly spent in open complex formation. In such a system, the outcome of fluctuations in RNA polymerase numbers (or transcription factors controlling closed complex formation) will depend on the switch’s present state. I.e. if the gene 2 is ‘ON’, the effects will be weak, but if it is gene 1 that is ‘ON’, the effects will be strong (more likely cause a switch in dynamics to occur). Future studies are needed to investigate how properties of genetic switches and genetic circuits are differentially sensitive to particular changes in the cellular composition.

Finally, we expect our results to be of value in the field of synthetic biology, which aims to engineer genetic networks with desired level of responsiveness to environmental cues by, among other, tuning the sensitivity to fluctuations in cellular component numbers at the single gene level. We expect our results to provide valuable information in this effort. For example, we believe that our results provide valuable clues on how to reduce present toggle switches’^44^ susceptibility to perturbations in cell physiology or in how to, alternatively, make the dynamics of a genetic circuit more responsive to changes in cellular physiology, in order to incorporate a cell’s current state into the circuit’s decision making process^13^.

## Materials and Methods

### Strains and plasmids

Experiments were conducted in *E. coli* strain DH5α-PRO, generously provided by I. Golding (Baylor College of Medicine, Houston, TX). It contains two genetic constructs: (a) pPROTet-K133 carrying P_*LtetO1*_-MS2d-GFP, and (b) a single-copy F-based vector, pIG-BAC with a P_*lac/ara-1*_ promoter controlling the production of mRFP1 followed by a 96 MS2d binding site array (P_*lac/ara-1*_-mRFP1-MS2d-96BS)^37^. We also use a modified system, with P_*lac*_ controlling the expression of an RNA with the 96 MS2d binding site array (named ‘P_*lac*_-MS2d-96BS’)^42^. Detailed information is provided in the supplementary information.

### Growth-conditions and microscopy

Cells were grown overnight at 30 °C with aeration and shaking in lysogeny broth (LB) medium, supplemented with appropriate antibiotics, diluted 1:1000 fold into fresh LB medium and allowed to grow at 37 °C at 250 RPM until an optical density of OD_600_ ≈ 0.3. Afterwards, a few µL of cells were placed between a 3% agarose gel pad and a glass coverslip, before assembling the FCS2 imaging chamber (Bioptechs). Cells were dispersed on the agarose gel pad, to give each the progeny of each cell enough space grow in numbers during the experiment. Prior to starting the experiment, the chamber was heated to 37 °C and placed under the microscope.

A flow of fresh (pre-warmed to 37 °C) LB medium containing the appropriate antibiotics was provided to cells under microscope observation by a peristaltic pump (Bioptechs) at a rate of 0.5 mL min^−1^. At first, cells were perfused with media for ~4 hours to grow colonies from individual cells. Next, we perfused the cells with 100 ng ml^−1^ anhydrotetracycline (aTc) to induce P_*LtetO1*_ for MS2d-GFP production. Finally, after 1 hour (usually, at this stage, each colony, i.e. lineage, reached a size of ~40 cells), we perfused cells with 1 mM IPTG (or 1% L-arabinose) and 100 ng ml^−1^ aTc.

Cells were visualized in a Nikon Eclipse (Ti-E, Nikon) inverted microscope with C2 + (Nikon), a point scanning confocal microscope system, using a 100x Apo TIRF (1.49 NA, oil) objective. Fluorescence images were acquired using a 488 nm argon ion laser (Melles-Griot) and a 514/30 nm emission filter (Nikon). The fluorescence images were acquired once per minute during the last 2 hours of the microscopy measurements. The laser shutter was open only during the exposure time to minimize photobleaching. Meanwhile, an external phase contrast system (Nikon) was used with a DS-Fi2 CCD camera (Nikon) to obtain phase contrast images once per every 5 minutes. All images were acquired with NIS-Elements software (Nikon).

### Data and image analysis

Data was analyzed using custom software written in MATLAB 2014a (MathWorks). Cells in phase contrast images were segmented using ‘CellAging’ (Fig. S1A)^45^. Alignment of the phase contrast images with the confocal images was done by selecting several landmarks in both images and using thin-plate spline interpolation for the registration transform. Fluorescent MS2d-GFP-RNA spots in each cell, at each frame, were detected with the Kernel Density Estimation (KDE) method using a Gaussian kernel (Fig. S1B)^46^. Cell background corrected spot intensities were then calculated by subtracting the mean cell background intensity multiplied by the area of the spots from the total fluorescence intensity of the spots. RNA numbers of individual cells at the different time moments as in^37^. From the distribution of background-corrected total spots intensity in cells, the first peak is set to correspond to the intensity of a single RNA molecule and the number of tagged RNAs in each spot is estimated by dividing its intensity by that of the first peak (Fig. S1C, Supplementary Information). To calculate the waiting times for the first production, the time intervals between consecutive production events and the total number of production events in lineages, the background-corrected total spots intensity over time in each cell was fitted to a monotone piecewise-constant function by least squares^46^. The number of terms was selected using the F-test with a p-value of 0.01. Each jump corresponds to the production of a single RNA (Fig. S1D). This method relies on the fact that, once tagged with MS2d-GFP, the RNA does not degrade and its fluorescence does not decay for several hours^39^. Waiting times for the first production of RNAs in each lineage were calculated by selecting cells without spots at the beginning of induction (i.e., without leaky expression), and detecting when the first production occurred in each branch of each lineage. Time intervals between consecutive RNA productions in individual cells were obtained by extracting the time between consecutive jumps in the total spots intensity (Fig. S1)^46^.

## Electronic supplementary material

Supplementary Material
