# Supplementary material for: Rate-limiting steps in transcription dictate sensitivity to variability in cellular components
Source: Sci Rep. 2017 Sep 6;7:10588. doi: 10.1038/s41598-017-11257-2 (PMC5587725; doi:10.1038/s41598-017-11257-2)
Supplement: Supplementary file 1 — Supplementary Material [file 41598_2017_11257_MOESM1_ESM.pdf]

Supplementary Material for: "Rate-limiting steps in transcription dictate sensitivity to variability in cellular components"

Jarno Mäkelä[1,2], Vinodh Kandavalli[1] and Andre S. Ribeiro[1,3,4,*]

[1]Laboratory of Biosystem Dynamics, BioMediTech Institute and Faculty of Biomedical Sciences and Engineering, Tampere University of Technology, 33101, Tampere, Finland.

[2]Present address: Department of Biochemistry, University of Oxford, South Parks Road, Oxford OX1 3QU, UK.

[3]Multi-scaled biodata analysis and modelling Research Community, Tampere University of Technology, 33101, Tampere, Finland.

[4]CA3 CTS/UNINOVA. Faculdade de Ciências e Tecnologia, Universidade Nova de Lisboa, Quinta da Torre, 2829-516, Caparica, Portugal.

*Correspondence: andre.ribeiro@tut.fi

Keywords: gene expression, in vivo single-RNA detection, lineage-to-lineage variability, rate-limiting steps in transcription

SI Materials and Methods

Strains and plasmids

The strain information of *E. coli* DH5α-PRO, generously provided by I. Golding (Baylor College of Medicine, Houston, TX) is: deoR, endA1, gyrA96, hsdR17(rK- mK+), recA1, relA1, supE44, thi-1, Δ(lacZYA-argF)U169, Φ80δlacZΔM15, F-, λ-, PN25/tetR, PlacIq/lacI, and SpR. It contains two genetic constructs: (a) pPROTet-K133 carrying P_{LtetO1}-MS2d-GFP, and (b) a single-copy F-based vector, pIG-BAC with a $P_{lac/ara\text{-}1}$ promoter controlling the production of mRFP1 followed by a 96 MS2d binding site array ($P_{lac/ara\text{-}1}$-mRFP1-MS2d-96BS) (Golding et al. 2005). We also use a modified system, with P_{lac} controlling the expression of an RNA with the 96 MS2d binding site array (named 'P_{lac}-MS2d-96BS')(Golding & Cox 2004). It was implemented in the same strain and uses the same reporter.

The strain produces necessary regulatory proteins for these constructs, namely, LacI, TetR and AraC, from the chromosome (Lutz & Bujard 1997). The MS2d-GFP single RNA detection system has been shown to detect individual target RNAs a few seconds after their transcription, provided that sufficient MS2d-GFP proteins are present in the cells (Golding & Cox 2004). Once tagged with MS2d-GFP, the RNA molecules do not degrade and their fluorescence does not decay significantly for a few hours (Tran et al. 2015). Also, it was shown that, in standard time-lapse microscopy measurements with consecutive images separate by 1 minute intervals, once appearing, each tagged RNA spot already exhibits 'full' fluorescence (Tran et al. 2015).

RNA numbers in cells

Estimation of RNA numbers in cells from the distribution of background-corrected total spots intensity in cells is only accurate if cells produce a small number of RNAs (note that the variability of each peak is expected to double from one peak to the next). We applied it here since this condition holds (see main manuscript). In this regard, these numbers are in agreement with several previous works. E.g., recently, RNA production *in vivo* from wild-type (WT) P_{lac}, $P_{lac\mathrm{UV5}}$ and a library of synthetic promoters was measured at the single molecule level using fluorescence *in situ* hybridization (FISH) (Jones et al. 2014). The mean mRNA numbers, under constitutive expression, varied between 0.04 and 10 per cell. E.g., WT P_{lac} exhibited a mean

mRNA number per cell of 0.4, while the stronger P_{lacUV5} had a mean RNA numbers per cell of 10 (Jones et al. 2014). Furthermore, it has been shown that the mean mRNA numbers per cell at the transcriptome level ranges from 10^{-4} to 10 mRNA per cell (Taniguchi et al. 2010). We find our measured RNA numbers to be in full accordance with these results.

qPCR

Target gene quantification was also done by qPCR. Cells containing the target plasmid were grown and induced with the respective inducers (1% arabinose for $P_{lac/ara-1}$-mRFP1-96BS, 10µM IPTG, 100µM IPTG and 1mM IPTG for $P_{lac/ara-1}$-mRFP1-96BS and 1mM IPTG for P_{lac}-lacZ-96BS) as described in the methods, and cells were harvested by centrifuging them at 8000×g for 5 minutes. To the pelleted cells, twice the amount of RNA protect reagent (Qiagen) was added and followed by enzymatic lysis with Tris EDTA lysozyme buffer (pH 8.0). The total RNA was isolated by the RNeasy kit (Qiagen) according to the manufacturer instructions and the concentration of RNA was quantified by a Nanovue plus spectrophotometer (GE Healthcare). The RNA samples were treated with DNase to remove residual DNA, followed by cDNA synthesis using the iSCRIPT reverse transcription super mix. The cDNA samples were mixed with the qPCR master mix containing iQ SYBR Green supermix (Biorad) with primers for the target and reference genes. The reaction was carried out in triplicates with a total reaction volume of 20 µl. For quantifying the target gene, we used mRFP1 primers (Forward: 5' TACGACGCCGAGGTCAAG 3' and Reverse: 5' TTGTGGGAGGTGATGTCCA 3'), and lacZ primers (Forward: 5' CCGGATCCTCGAGAGCTTAG 3' and Reverse: 5' CTAATCGATTCAATTGGGTAACG 3'). For the reference gene, we used 16S RNA primers (Forward: 5' CGTCAGCTCGTGTTGTGAA 3' and Reverse: 5' GGACCGCTGGCAACAAAG 3') were used. The qPCR experiments were performed by a MiniOpticon Real time PCR system (Biorad). The following conditions were used during the reaction: 40 cycles of 95°C for 10 s, 52°C for 30 s and 72°C for 30 s for each cDNA replicate. We used no-RT controls and no-template controls to crosscheck non-specific signals and contamination. PCR efficiencies of these reactions were greater than 95%. The data from CFX Manager TM Software was used to calculate the relative gene expression and its standard error (Livak & Schmittgen 2001).

Western Blotting

Cultures of *E. coli* DH5α-PRO strain were grown in media of different richness ("1x", "0.5x" and "0.25x"), as described in materials and methods. Cells were harvested at OD_{600} of 0.3 and lysed with the B-PER bacterial protein extraction reagent (Thermo scientific) in the presence of protease inhibitors for 10 min. Subsequently, the lysed cells were centrifuged at 15000×g for 10 mins, supernatants collected, diluted in the 4X laemmli sample loading buffer containing β-mercaptoethanol and boiled for 5 mins at 95 °C. The samples from all the cultures, each containing ~30 µg of total soluble proteins, were resolved by 4 to 20 % TGX stain free precast gels (Biorad). Proteins were separated by electrophoresis and then electro-transferred to the PVDF membrane. Membranes were blocked with 5% non-fat milk and incubated with respective primary RpoC antibodies of 1:2000 dilutions (Biolegend) overnight at 4 °C, followed by the appropriate HRP-secondary antibodies 1:5000 dilutions (Sigma Aldrich) for 1 h at room temperature. For detection, chemilumiscence reagent (Biorad) was used. Images were generated by the Chemidoc *XRS* system (Biorad) (Fig. S12). Band intensity quantification was done by the Image lab software (version 5.2.1).

τ-plot

Mean transcription rates (i.e. mean RNA production rates) depend on the free RNAp concentration of the cells (McClure 1985; Liang et al. 1999; Ehrenberg et al. 2013). These concentrations can be tuned by altering media composition in a specific manner (Liang et al. 1999; Patrick et al. 2015). This was achieved in (Lloyd-Price et al. 2016) by modifying components of LB media. It was further shown that in a certain range of media compositions, the relative free RNAp concentration can be assessed from the total RNAp concentration, because it varies in a linear fashion with the changes in media composition (Lloyd-Price et al. 2016). Also, no evidence for factors other than the free RNAp concentration affecting the rate of production from target promoter was found. Following this methodology, we used media compositions per 100 ml as follows: ("0.25x" condition) 0.25 g tryptone, 0.125 g yeast extract and 1 g NaCl (pH 7.0); ("0.5x" condition) 0.5 g tryptone, 0.25 g yeast extract and 1 g NaCl (pH 7.0); ("1x" condition) 1 g tryptone, 0.5 g yeast extract and 1 g NaCl (pH 7.0). The relative RNAp concentrations in each condition were assessed by measuring the level of the RpoC protein by Western blot (Fig. S12). These measurements confirmed that the relative RNAp levels change

linearly with media compositions, as reported in (Lloyd-Price et al. 2016). For each condition, the target RNA production rates in different media were measured by qPCR.

Next, based on the premise that the RNAp concentration only affects the duration of closed complex formation but not the duration of the subsequent rate-limiting steps (Liang et al. 1999; Lloyd-Price et al. 2016), we extracted the ratio between the RNAp-dependent fraction (τ_{cc}) of the mean duration of the time intervals between transcription events and the overall mean duration of the time intervals between transcription events (Δt), $\tau_{cc}/\Delta t$, following the methodology proposed in (Liang et al. 1999; Lloyd-Price et al. 2016). I.e., the inverse of RNA production rates were plotted against the inverse of relative RNAp levels and fitted by a line using weighted total least squares (Krystek & Anton 2007). From this fit, $\tau_{cc}/\Delta t$ was estimated by extrapolating the inverse of RNA production rate to an infinite RNAp concentration (Lloyd-Price et al. 2016; Liang et al. 1999; Patrick et al. 2015). The results are shown in (Fig. S13).

Stochastic model of gene activation and transcription

The stochastic model considers both gene activation following the appearance of inducers in the media, and transcription following the activation step. Gene activation is the process by which a gene that is in a non-producing state enters a producing state, via a multi-step process. This process includes events such as diffusion of the activator molecules in the periplasm and cytoplasm, binding to a transcription factor, protein-protein interactions etc. These events differ with the induction system of each particular gene (Schleif 2000; Megerle et al. 2008; Skerra 1994; Weickert, M.J. and Adhya 1993).

In the case of the promoters studied in this work, the waiting times for gene activation by the external inducers have been measured at the single cell level and shown to exhibit dynamics of activation of the target gene that can be well modelled by a 2-step stochastic process (Fig. 1B) (Mäkelä et al. 2013; Megerle et al. 2008; Fritz et al. 2014; Tran et al. 2015). The first-passage time distribution, which corresponds to the total time spent in each of the states of the process, can be thus described by a general model of the form (Mäkelä et al. 2013; Moffitt & Bustamante 2014):

$$I_1 \xrightarrow{Upt \cdot k_1} I_2 \xrightarrow{Upt \cdot k_2} S_0 \quad [1]$$

Here, I_1 is the non-producing state of the system, I_2 is an intermediate state, and S_0 is the producing state of the system, in which the promoter is available for transcription. These

reactions occur at rates k_1 and k_2, respectively, and are both catalyzed by an uptake protein (Upt). These rates correspond to the total time spent on each state (Moffitt & Bustamante 2014). It is noted that the number of uptake proteins is set to affect the rates of both steps, as the shape of the distribution was found not to change with inducer concentration (Megerle et al. 2008).

Note that the dependence of the reactions on the numbers of Upt proteins allows the model to exhibit cell-to-cell diversity in the kinetics of gene activation, provided that these numbers differ between cells (similarly, the dynamics could differ over time).

For parameter values, we made use of measurements of the arabinose utilization system, which has been reported to take, on average, ~23 min to activate each cell, with a standard deviation of ~10 min (Megerle et al. 2008). As an independent validation, we used a plate reader to measure the production kinetics over time following induction (Fig. S9). Given the maturation time of RFP1 of 0.7 h (Campbell et al. 2002), the results are in agreement with the single RNA measurements.

It is worth noting that previous studies on gene activation (Johnson & Schleif 1995; Daruwalla et al. 1981) reported faster kinetics than those measured here and also reported in (Mäkelä et al. 2013). This is likely due to several reasons. Namely, as mentioned in the main manuscript, first, different strains were used. E.g., DH5α-PRO, the strain used here, was modified to contain a very high copy number of lac repressors (~3000 vs. ~20 in wild type) (Lutz & Bujard 1997). Second, in the case of the $P_{lac/ara\text{-}1}$ promoter, note that our cells do not code for lactose permease, which transports lactose into the cell. Finally, for the case of induction with arabinose, we do not de-repress the promoter with IPTG, which is expected to delay RNA production significantly. We note that, aside from the reduced speed in RNA production, we do not expect these differences to cause additional significant functional differences.

Next, we describe the process of active transcription also included in the model. In *E. coli*, this process consists of a sequence of steps, with the formations of the closed complex and open complex being, in most promoters, the rate-limiting ones (McClure 1985; Saecker et al. 2011; Lutz et al. 2001). Transcription, as a dynamic process, can thus be formulated as (McClure 1985):

$$\mathrm{RNAp} + \mathrm{Pro} \xleftrightarrow{K_B} \mathrm{RP_c} \xrightarrow{k_f} \mathrm{RP_o} \rightarrow\rightarrow\rightarrow \mathrm{RNA} \qquad [2]$$

where transcription initiates by RNA polymerase holoenzyme (RNAp) binding to a promoter (Pro) and forming the closed complex (RP_c). This step is usually reversible. Following several

attempts, the holoenzyme will eventually succeed in opening the DNA strands, thus creating a transcription bubble, and assemble the polymerase clamp through several intermediate steps to form a stable open complex (RP_o). Finally, the holoenzyme will form an elongation complex and synthesize the nascent RNA molecule.

From [2], it is possible to extract a time interval distribution between transcription events (Δt). For this, we use the fact that the first-passage time distribution to produce an RNA is observationally equivalent to the distribution described by a model of the form (Lloyd-Price et al. 2016; Moffitt & Bustamante 2014):

$$S_0 \xrightarrow{R\cdot k_3} S_1 \xrightarrow{k_4} S_2 \xrightarrow{k_5} S_0 + \mathrm{RNA} \qquad [3]$$

Here, S_0 is a state in which the promoter is available for transcription (following induction). Transition to state S_1 occurs at the rate $R \cdot k_3$ (R being the number of RNAp molecules) and, following this, transition to state S_2 occurs at the rate k_4. Finally, an RNA is produced and the promoter returns to state S_0 at the rate k_5. RNA degradation is modelled as an exponential process with a rate of 5 min^{-1} (Bernstein et al. 2002; Chen et al. 2015). Models of this form have been shown to fit recent *in vivo* measurements of Δt at the single RNA level (see e.g. (Lloyd-Price et al. 2016; Tran et al. 2015)).

A recent study has quantified that, for $P_{lac/ara\text{-}1}$, the RNAp-dependent fraction of time of transcription initiation ($R^{-1} \cdot k_3^{-1}$) lasts ~788 s, while the non-RNAp dependent fraction lasts ~193 s (k_4^{-1}) (Lloyd-Price et al. 2016). The RNAp dependent stage of initiation ($R^{-1} \cdot k_3^{-1}$) includes the reversible closed complex formation and transcriptionally inactive promoter states, which occur, e.g., due to binding and unbinding of the repressor (Lutz et al. 2001) and accumulation of negative supercoiling in the DNA (Chong et al. 2014).

Meanwhile, the steps following open complex formation have been found to be fast (here, this is modeled by setting $k_5 = \infty$), indicating that abortive initiation events do not play a major role in the dynamics of RNA production in $P_{lac/ara\text{-}1}$ (Lloyd-Price et al. 2016). This is expected since only in rare promoters, whose open complexes exhibit extremely short half-lives, is promoter escape expected to be rate-limiting (Hsu 2002).

In (Taniguchi et al. 2010), a global characterization of cell-to-cell variability in protein numbers showed a noise limit that is independent of the mean. The distribution of protein

numbers in a population was found to be well fitted by a discrete negative binomial distribution (Taniguchi et al. 2010). Here, we model the variability in uptake protein numbers (for the induction process) and RNAp (for the transcription process) taking this into account. Parameter values for the negative binomial distribution for the RNAp variability ($CV^2 = 0.1$) were obtained from (Jones et al. 2014; Taniguchi et al. 2010) and for the uptake protein ($CV^2 = 0.27$) from (Megerle et al. 2008).

The variability in RNAp numbers affects the rate of closed complex formation (McClure 1980; McClure 1985; Saecker et al. 2011). The variability in uptake protein numbers affects the rates of both steps in initiation, as the shape of the distribution does not change with inducer concentration (Megerle et al. 2008). Since fluctuations in protein numbers were shown to have a time scale of several cell cycles (up to 5 hours) (Taniguchi et al. 2010; Hensel et al. 2012; Rosenfeld et al. 2005), we assume fixed protein numbers for each cell in the models (but differing between cells as noted above).

CME solution

To predict the time-varying probability distributions from the models, we make use of a direct integration of the Chemical Master Equation (CME) of the sum of d-exponential variates model for gene activation and transcription using the Finite State Projection algorithm (Munsky & Khammash 2006). This method truncates the infinite state space of the CME, so that the amount of probability outside the truncated region is negligible, and formulates a finite set of linear ordinary differential equations for each possible state of the system. The state space was truncated at 100 RNA molecules. This means that this space contains virtually all of the total probability in the system (we never observed a cell to have more than 20 RNAs). The probability mass vector at each time moment is then obtained for all phenotypes. Next, the population distribution is obtained by utilizing the negative binomial distribution to assign weight for each combination of molecule numbers. From this distribution, we calculate mean and variance of RNA molecules between phenotypes at each time moment.

Fitting empirical distributions to a sum of d-exponential variates

To fit an empirical distribution to a sum of d-exponential variates (of possibly unequal rates), as in (Mäkelä et al. 2013), we select the exponential rate parameters $\lambda_1,...,\lambda_d$ so that the

Kolmogorov-Smirnov (K-S) statistics is minimized. I.e., parameters are selected as $\hat{\theta} = \arg\max_{\theta=\lambda_1,\ldots,\lambda_d} \sup_x \left|F_\theta(x) - G(x)\right|$, where $F_\theta(x)$ is the cumulative distribution function (CDF) of a sum of *d* exponentials with parameters $\theta = (\lambda_1,\ldots,\lambda_d)$, and $G(x)$ is the CDF of the empirical distribution.

$$F_{\theta=L_1,\ldots,L_d}(x) := \sum_{i=1}^{d}\left(\left(1-e^{-L_i x}\right)\prod_{\substack{j=1\\ j\neq i}}^{d}\frac{L_j}{L_j - L_i}\right) \quad [4]$$

The parameter values θ are found using a nonlinear numerical optimizer. This method is convenient, since if the K-S test is rejected for the parameters $\hat{\theta}$, it would also be rejected for any other set of parameters θ in this family of fitted distributions, indicating that these distributions are inappropriate models of the data.

Supplementary Figures

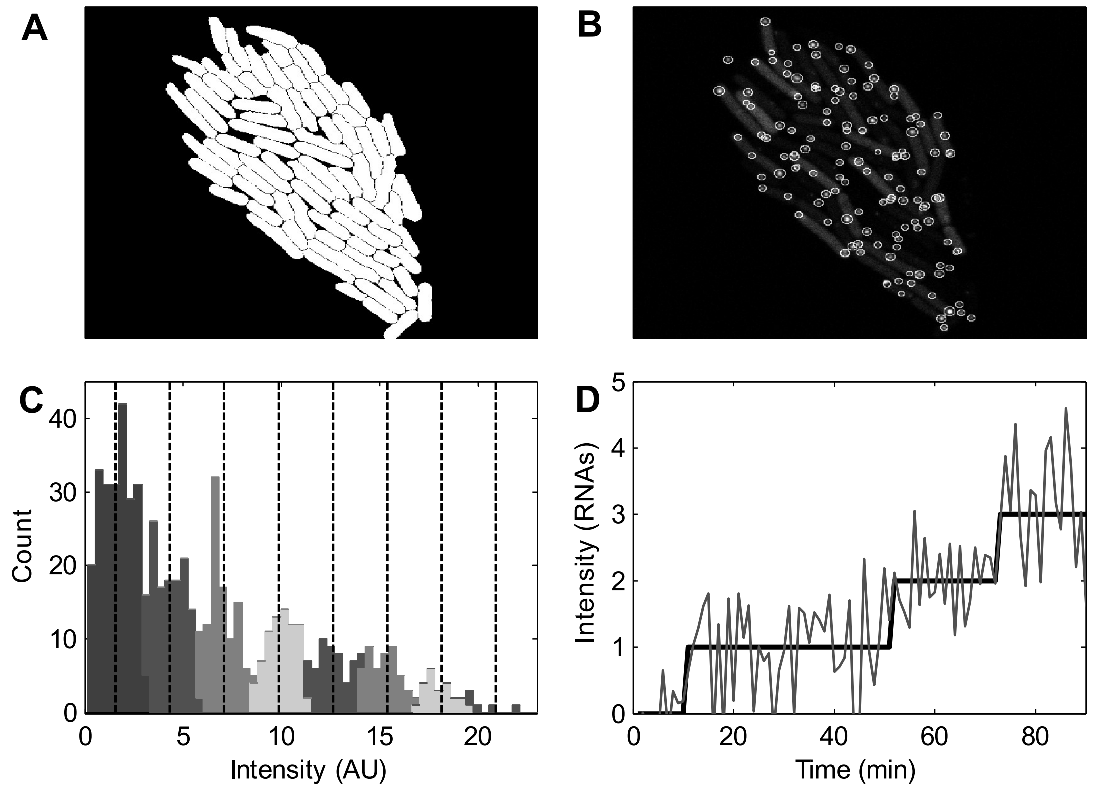

Figure S1. Image analysis and RNA quantification. **(A)** Segmented cell backgrounds **(B)** Detection of RNA spots using the Kernel Density Estimation. **(C)** Analysis of RNA numbers from single cells. **(D)** Example of the results of the method of detection of novel RNA appearance events in a single cell from time series data on total RNA-spot fluorescence in a cell.

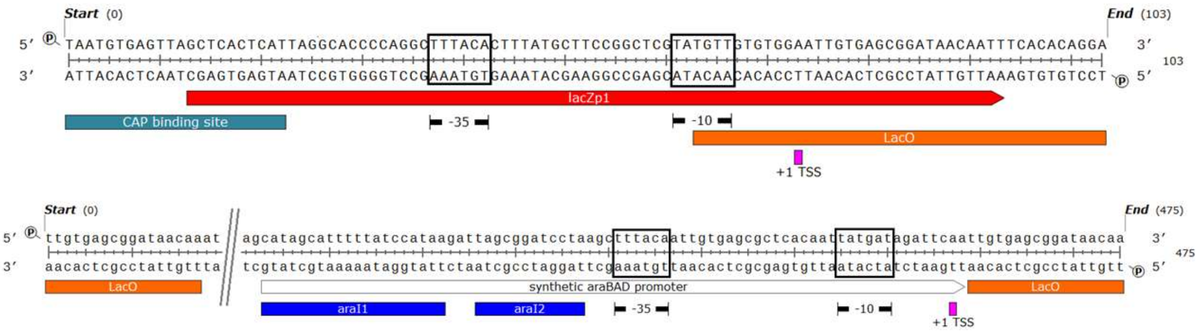

Figure S2. Topography and sequences of promoters (top) P_{lac} (Golding & Cox 2004) and (bottom) $P_{lac/ara\text{-}1}$ (Lutz & Bujard 1997). RNA polymerase binding sites are boxed. The two small pink boxes show the transcriptional start site. Blue boxes show the operator binding sites of araI1 and araI2, and orange boxes show the operator sites of lacO. White and red arrows show the araBAD and the LacZp1 promoters, respectively. The figures were produced using the SnapGene Software (GSL Biotech, Chicago, IL, USA).

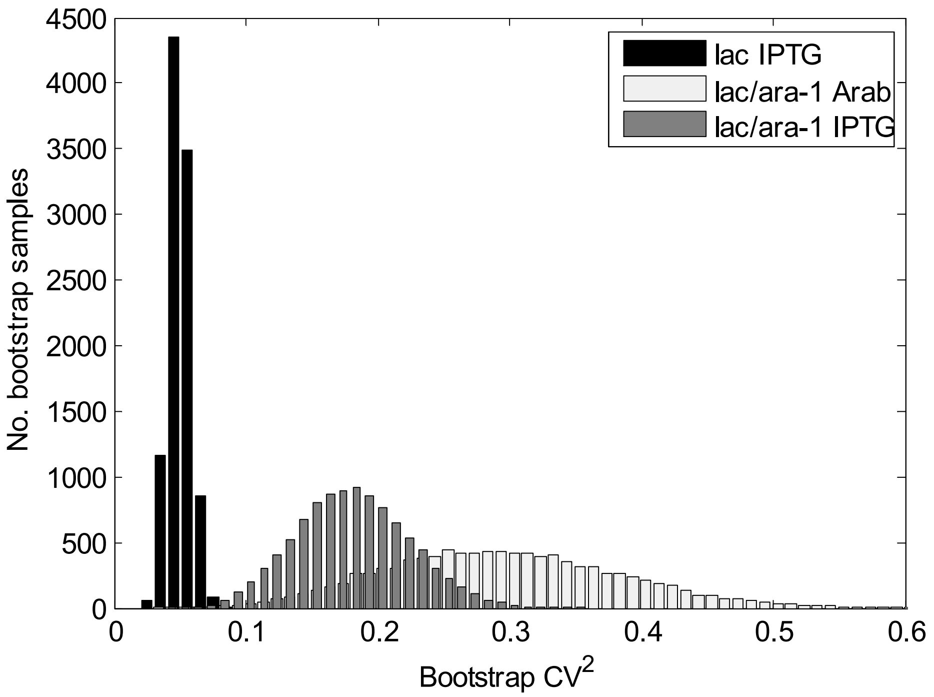

Figure S3. Bootstrapping of cells in the lineages. CV^2_{phe} of the RNA numbers between lineages. Std of bootstrapping samples corresponding to standard errors for lac IPTG, lac/ara-1 IPTG and lac/ara-1 Arab are 0.008, 0.043 and 0.089, respectively.

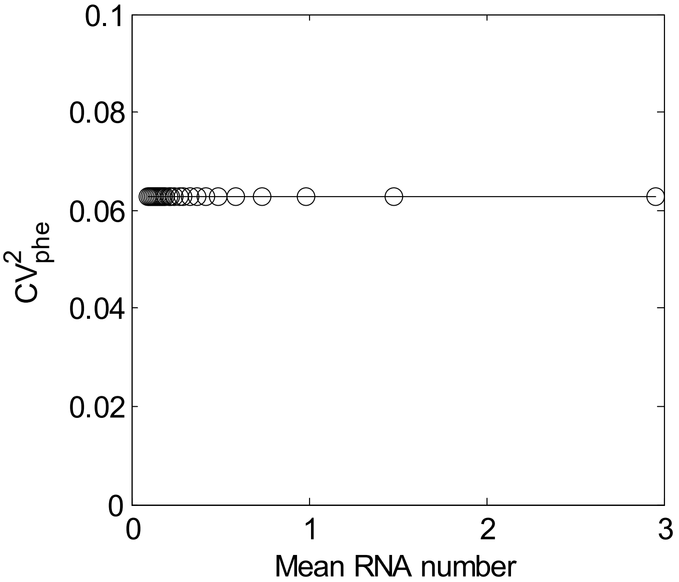

Figure S4. CV^2_{phe} as a function of mean RNA number. Different mean RNA numbers were achieved by changing the overall duration of transcription. Time-lengths between consecutive transcription events were modeled to be between 100 s and 3000 s, resulting in different mean RNA levels (while maintaining constant the ratio $\tau_{cc}/\Delta t$). From these results, we conclude that CV^2_{phe} of RNA numbers is independent of the mean production rate of that RNA.

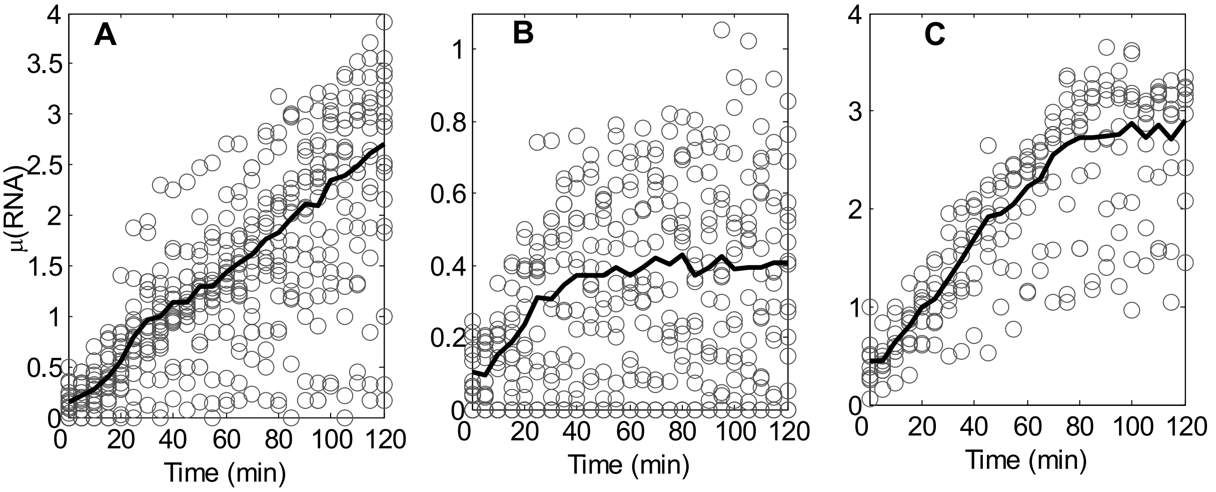

Figure S5. Mean RNA numbers per cell in each lineage over time, following induction. **(A)** P*lac/ara-1* induced with 1 mM IPTG (1468 cells). **(B)** P*lac/ara-1* induced with 1 % L-arabinose (1296 cells). **(C)** P*lac* induced with 1 mM IPTG (1665 cells). The degree of lineage-to-lineage variability in each condition is expected to be a consequence of, among other causes, the lineage-to-lineage variability in cellular components affecting the kinetics of active transcription and inducers intake (Mäkelä et al, 2013). The contribution on the variability from these two processes is also expected to change over time in each condition, and seems to differ between the 3 conditions.

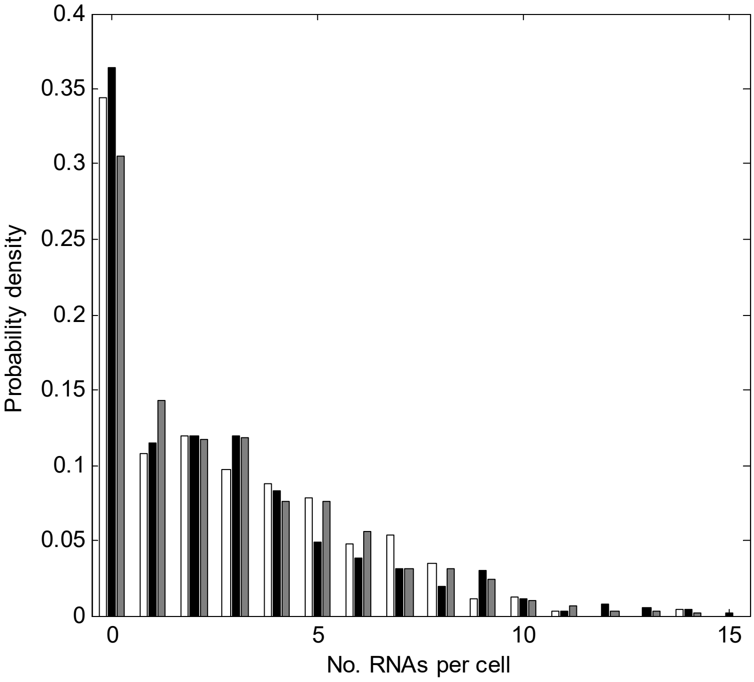

Figure S6. RNA numbers in individual cells after 2 hours of induction from 3 independent experiments of $P_{lac/ara\text{-}1}$ induced with IPTG. Experiment 1 is a time series measurement and experiments 2 and 3 are cell population measurements. To compare the RNA distributions from different experiments, we used the two-sample Kolmogorov-Smirnov test to the test the null hypothesis that the samples are drawn from the same distribution. We obtained p-values of 0.24 (between experiments 1 and 2) and 0.58 (between experiments 1 and 3) and, thus, the null hypothesis cannot be rejected (for p-value < 0.01, it is generally accepted that the hypothesis that the two distributions are the same should be rejected). The number of cells observed in experiments 1, 2 and 3 were 924, 1219 and 764, respectively. No statistically significant differences between the experiments are visible.

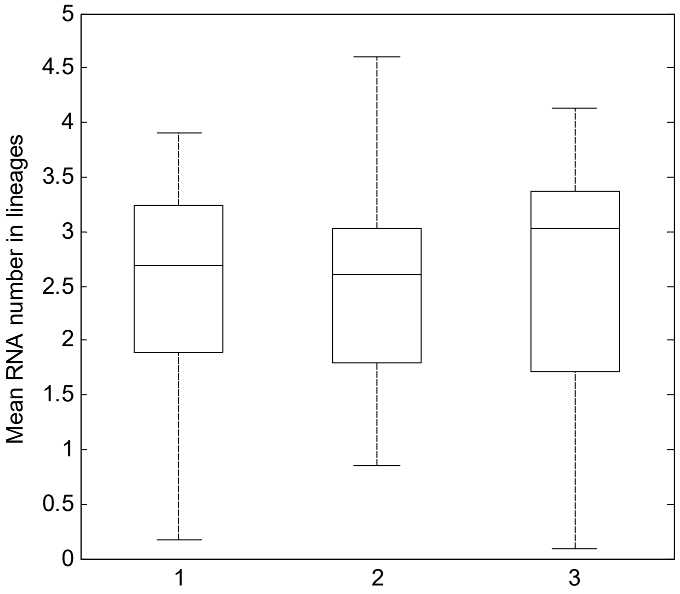

Figure S7. Independent measurements of lineage-to-lineage variability in mean RNA numbers for $P_{lac/ara-1}$ after 2h of induction by 1mM IPTG. Experiment 1 is a time series and experiments 2 and 3 are cell population measurements. We show the boxplots of mean RNA numbers for each experiment. To test the null hypothesis that the samples are drawn from the same distribution, we used the two-sample Kolmogorov-Smirnov test and obtained p-values of 0.77 (experiments 1 and 2) and 0.82 (experiments 1 and 3). For p-value < 0.01 it is generally accepted that the hypothesis that the two distributions are the same should be rejected. Given this, the null hypothesis cannot be rejected. In all conditions, the variability between lineages in mean RNA numbers is above chance. Relevantly, this variability differs with the promoter as well as with the inducer.

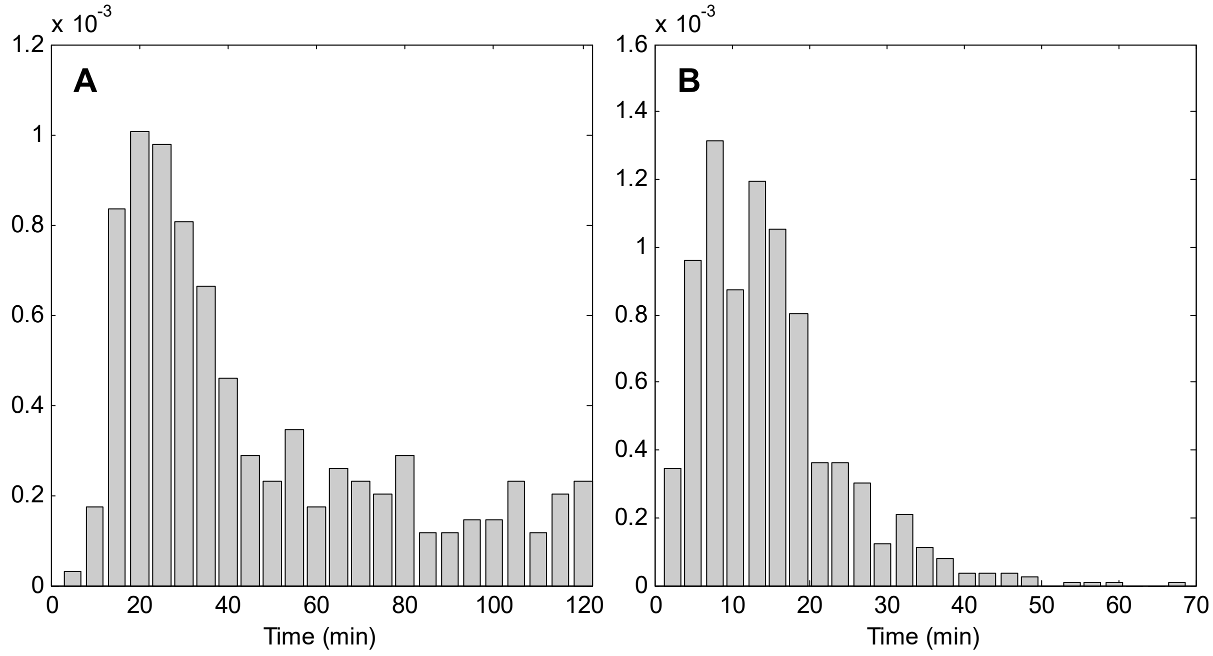

Figure S8. Production kinetics of RNAs by $P_{lac/ara\text{-}1}$ induced with IPTG. **(A)** Lineage waiting times for the first production event, and **(B)** time intervals between consecutive production events in individual cells. The y-axis is the probability density.

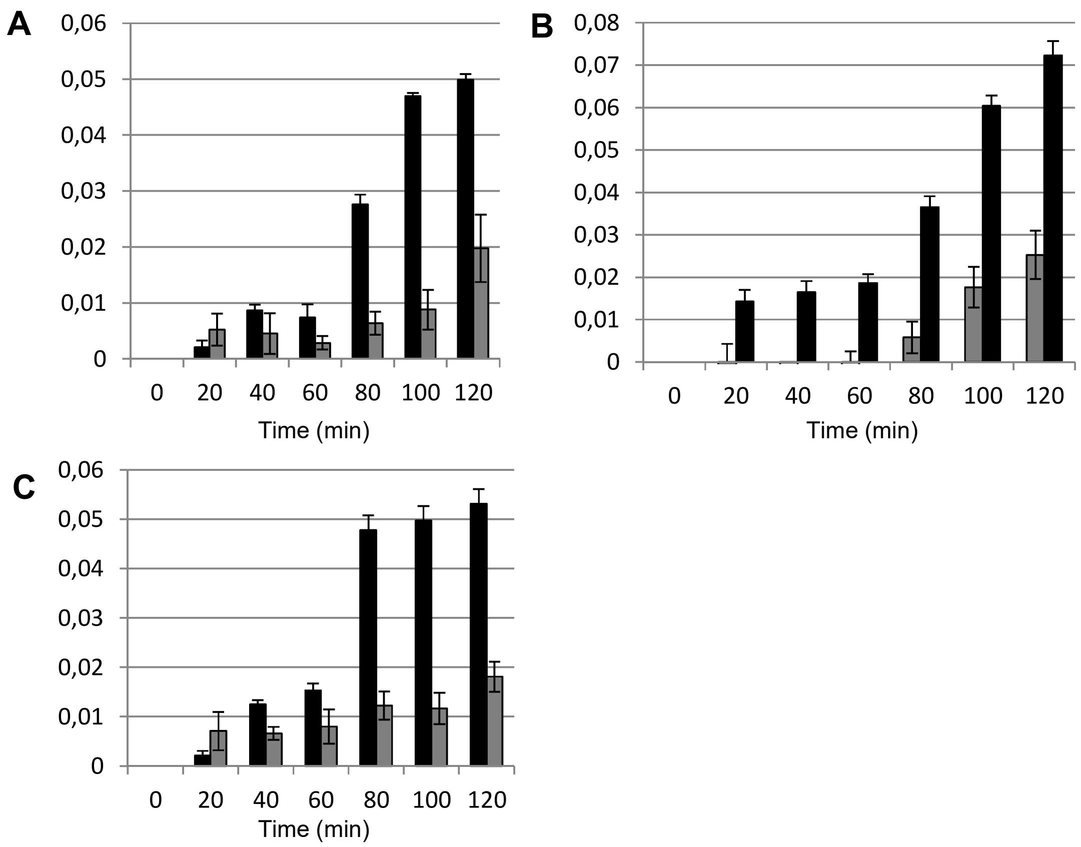

Figure S9. Plate reader measurements of target promoter expression. **(A)** $P_{lac/ara\text{-}1}$ induced with 1mM IPTG (black). **(B)** $P_{lac/ara\text{-}1}$ induced with 1% l-arabinose (black). **(C)** P_{lac} induced with 1mM IPTG (black). The grey bar is the control (without induction). Data is normalized with the first time moment of the times series. y-axis is the normalized fluorescence intensity.

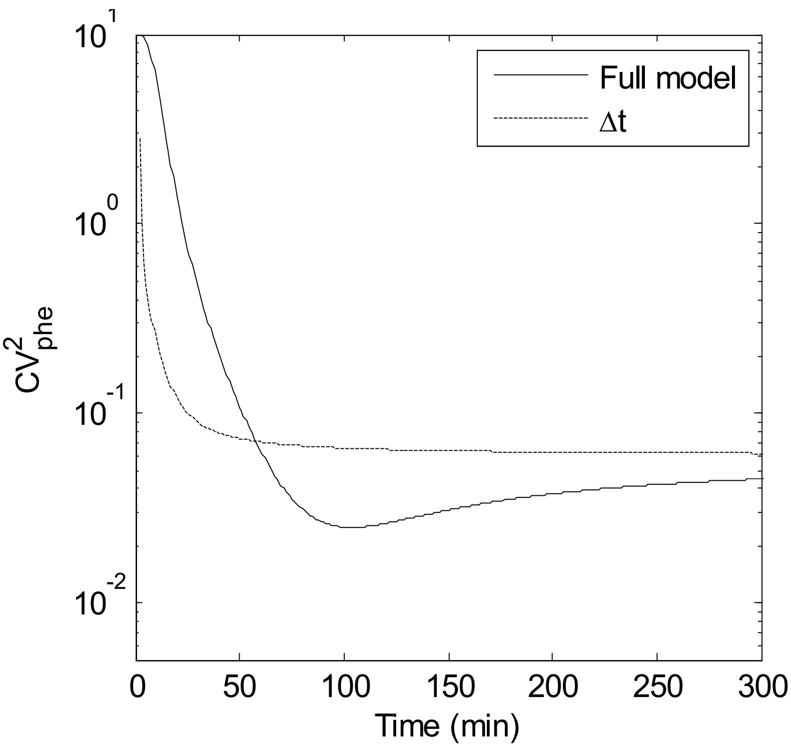

Figure S10. CV^2_{phe} for models of $P_{lac/ara-1}$ induced with arabinose. The models consist of both processes (Full model: Δt and t_0), and only the Δt process. Over time, the CV^2_{phe} of the full model and the CV^2_{phe} of the model accounting for active transcription (Δt process) become similar, as the activation events become more rare as time progresses.

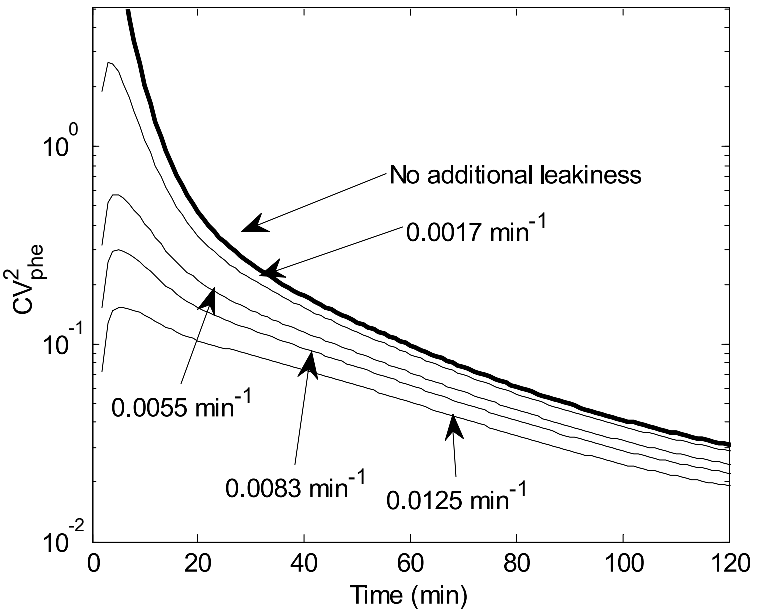

Figure S11. Model of $P_{lac/ara-1}$ induced with IPTG (full model with different rates of leakiness). Leakiness is modeled as an extra reaction of production whose dynamics is that of a Poisson process (see rates of leakiness in each case). Visibly, increasing the rate of leakiness decreases the lineages' CV^2_{phe}.

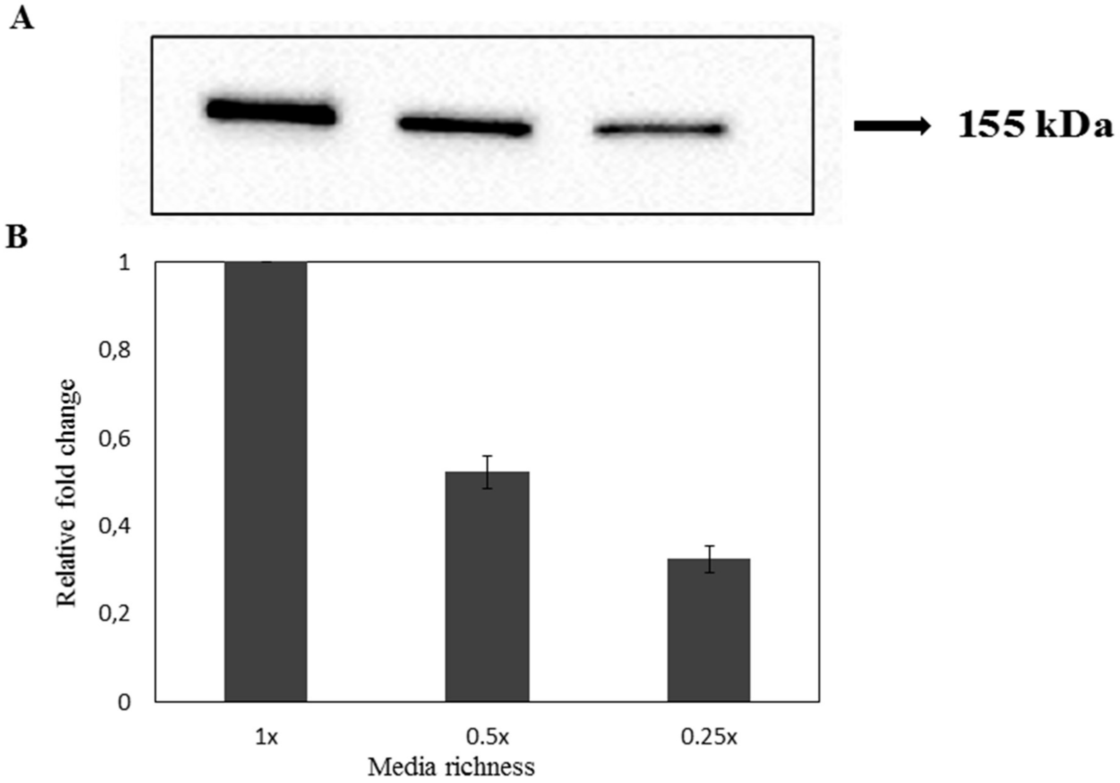

Figure S12. Relative RpoC protein levels of *E. coli* cells of the strain DH5α-PRO when grown in different media richness (1x, 0.5x, and 0.25x) measured by Western blotting. **(A)** A replicate of Western blot image. **(B)** The observed levels of the RpoC protein compared to the protein level in 1x medium (treated as a 100% reference) were 52% in 0.5x medium and 32% in 0.25x medium. The error bars (0.5x: 3.8%, 0.25x: 3.1%) reflect 90% confidence intervals in the differences for 4 biological replicates. The relative band intensities were quantified by the Image lab software (version 5.2.1) from the chemiluminescence blots. We find large differences in RpoC protein levels for different media richness.

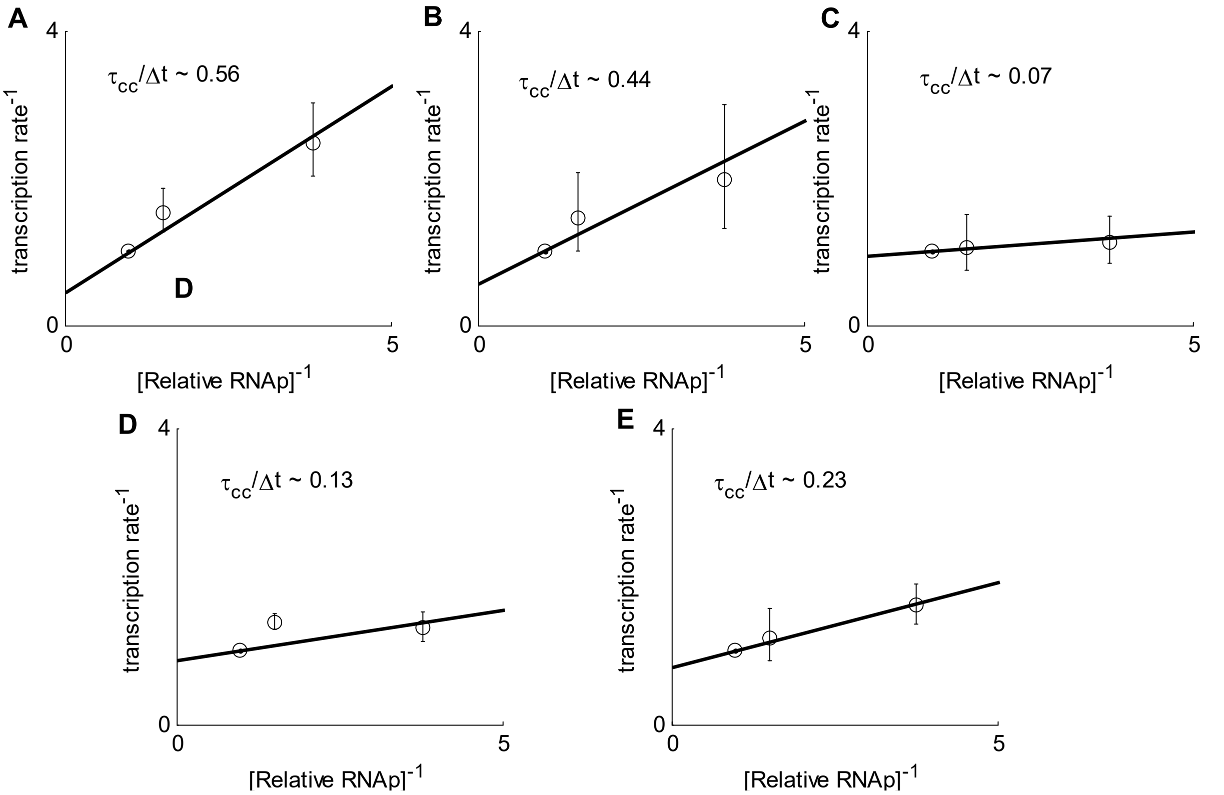

Figure S13. τ-plots. **(A)** $P_{lac/ara-1}$ with 1 mM IPTG. **(B)** $P_{lac/ara-1}$ with 100 μM IPTG. **(C)** $P_{lac/ara-1}$ with 10 μM IPTG. **(D)** P_{lac} with 1 mM IPTG. **(E)** $P_{lac/ara-1}$ with 1 % Arabinose. Also shown is the resulting ratio $\tau_{cc}/\Delta t$ in each case. Visibly, $\tau_{cc}/\Delta t$ differs with inducer concentration (compare A, B, and C results), type of inducer (compare, e.g., A and E), and between different promoters (compare A and D).

Supplementary Tables

Table S1. Correlation between the distance to the center of a colony of a cell and its number of RNA molecules in each of the three conditions studied. We calculated the correlation between the distance to colony center and the number of produced RNA molecules in individual cells to assess whether the extracellular environment (i.e. inducer concentration or media richness) had sufficient local variability in conditions to generate tangible differences in the RNA production rates of individual cells. In all conditions, there are only weak, not statistically significant correlations, indicating that the induction level of individual cells is not location-dependent.

	ρ	p-value
$P_{lac/ara\text{-}1}$ IPTG	0.0394	0.27
$P_{lac/ara\text{-}1}$ Arab	-0.0230	0.59
P_{lac} IPTG	-0.0175	0.63

Table S2. Measured mean values (μ) and CV^2_{phe} of t_{act} and Δt for $P_{lac/ara\text{-}1}$ with IPTG (15 lineages), $P_{lac/ara\text{-}1}$ with arabinose (10 lineages), and P_{lac} with IPTG (8 lineages). Also shown is the leakiness (percentage of cells with RNAs prior to induction) and the total number of RNA production events observed. Error estimates are from bootstrap sampling of the cells in the lineages. The CV^2_{phe} in t_{act} as well as in Δt between lineages differ between all conditions.

	$P_{lac/ara\text{-}1}$ IPTG	$P_{lac/ara\text{-}1}$ Arab	P_{lac} IPTG
No. RNA prod. events	1799	391	1388
Leakiness	9.8%	7.2%	34.3%
μ (t_{act}) (s)	2030 ± 191	2488 ± 209	1085 ± 126
CV^2_{phe} (t_{act})	0.141 ± 0.041	0.078 ± 0.023	0.124 ± 0.056
μ (Δt) (s)	889 ± 25	1254 ± 85	1365 ± 39
CV^2_{phe} (Δt)	0.014 ± 0.004	0.051 ± 0.013	0.008 ± 0.004

References

Bernstein, J.A. et al., 2002. Global analysis of mRNA decay and abundance in Escherichia coli at single-gene resolution using two-color fluorescent DNA microarrays. *Proceedings of the National Academy of Sciences of the United States of America*, 99(15), pp.9697–9702.

Campbell, R.E. et al., 2002. A monomeric red fluorescent protein. *Proceedings of the National Academy of Sciences of the United States of America*, 99(12), pp.7877–7882.

Chen, H. et al., 2015. Genome-wide study of mRNA degradation and transcript elongation in Escherichia coli. *Molecular Systems Biology*, 11(781), pp.1–11.

Chong, S. et al., 2014. Mechanism of Transcriptional Bursting in Bacteria. *Cell*, 158(2), pp.314–326.

Daruwalla, K.R., Paxton, A.T. & Henderson, P.J.F., 1981. Energization of the transport systems for arabinose and comparison with galactose transport in Escherichia coli. *Biochemical Journal*, 200(3), pp.611–627.

Ehrenberg, M., Bremer, H. & Dennis, P.P., 2013. Medium-dependent control of the bacterial growth rate. *Biochimie*, 95(4), pp.643–658.

Fritz, G. et al., 2014. Single cell kinetics of phenotypic switching in the arabinose utilization system of E. coli. *PLoS ONE*, 9(2), p.e89532.

Golding, I. et al., 2005. Real-time kinetics of gene activity in individual bacteria. *Cell*, 123(6), pp.1025–1036.

Golding, I. & Cox, E.C., 2004. RNA dynamics in live Escherichia coli cells. *Proceedings of the National Academy of Sciences of the United States of America*, 101(31), pp.11310–11315.

Hensel, Z. et al., 2012. Stochastic expression dynamics of a transcription factor revealed by single-molecule noise analysis. *Nature Structural and Molecular Biology*, 19(8), pp.797–802.

Hsu, L.M., 2002. Promoter clearance and escape in prokaryotes. *Biochimica et Biophysica Acta*, 1577(2), pp.191–207.

Johnson, C.M. & Schleif, R.F., 1995. In vivo induction kinetics of the arabinose promoters in Escherichia coli. *Journal Of Bacteriology*, 177(12), pp.3438–3442.

Jones, D.L., Brewster, R.C. & Phillips, R., 2014. Promoter architecture dictates cell-to-cell variability in gene expression. *Science*, 346(6216), pp.1533–1537.

Krystek, M. & Anton, M., 2007. A weighted total least-squares algorithm for fitting a straight line. *Measurement Science and Technology*, 18, pp.3438–3442.

Liang, S. et al., 1999. Activities of constitutive promoters in Escherichia coli. *Journal of molecular biology*, 292(1), pp.19–37.

Livak, K.J. & Schmittgen, T.D., 2001. Analysis of relative gene expression data using real-time quantitative PCR and the 2-DDCT method. *Methods*, 25(4), pp.402–408.

Lloyd-Price, J. et al., 2016. Dissecting the stochastic transcription initiation process in live Escherichia coli. *DNA Research*, 23(3), pp.203–214.

Lutz, R. et al., 2001. Dissecting the functional program of Escherichia coli promoters: the combined mode of action of Lac repressor and AraC activator. *Nucleic Acids Research*, 29(18), pp.3873–3881.

Lutz, R. & Bujard, H., 1997. Independent and tight regulation of transcriptional units in Escherichia coli via the LacR/O, the TetR/O and AraC/I1-I 2 regulatory elements. *Nucleic Acids Research*, 25(6), pp.1203–1210.

McClure, W.R., 1985. Mechanism and control of transcription initiation in prokaryotes. *Annual Review of Biochemistry*, 54, pp.171–204.

McClure, W.R., 1980. Rate-limiting steps in RNA chain initiation. *Proceedings of the National Academy of Sciences of the United States of America*, 77(10), pp.5634–5638.

Megerle, J.A. et al., 2008. Timing and dynamics of single cell gene expression in the arabinose utilization system. *Biophysical Journal*, 95(4), pp.2103–2115.

Moffitt, J.R. & Bustamante, C., 2014. Extracting signal from noise: Kinetic mechanisms from a Michaelis-Menten-like expression for enzymatic fluctuations. *FEBS Journal*, 281(2), pp.498–517.

Munsky, B. & Khammash, M., 2006. The finite state projection algorithm for the solution of the chemical master equation. *Journal of Chemical Physics*, 124(4), p.44104.

Mäkelä, J. et al., 2013. In vivo single-molecule kinetics of activation and subsequent activity of the arabinose promoter. *Nucleic Acids Research*, 41(13), pp.6544–6552.

Patrick, M. et al., 2015. Free RNA polymerase in E. coli. *Biochimie*, 119, pp.80–91.

Rosenfeld, N. et al., 2005. Gene regulation at the single-cell level. *Science*, 307(5717), pp.1962–1965.

Saecker, R.M., Record, M.T. & DeHaseth, P.L., 2011. Mechanism of Bacterial Transcription

Initiation: RNA Polymerase - Promoter Binding, Isomerization to Initiation-Competent Open Complexes, and Initiation of RNA Synthesis. *Journal of Molecular Biology*, 412(5), pp.754–771.

Schleif, R., 2000. Regulation of the L-arabinose operon of Escherichia coli. *Trends in Genetics*, 16(12), pp.559–565.

Skerra, A., 1994. Use of the tetracycline promoter for the tightly regulated production of a murine antibody fragment in Escherichia coli. *Gene*, 151(1–2), pp.131–135.

Taniguchi, Y. et al., 2010. Quantifying E. coli proteome and transcriptome with single-molecule sensitivity in single cells. *Science*, 329(5991), pp.533–538.

Tran, H. et al., 2015. Kinetics of the cellular intake of a gene expression inducer at high concentrations. *Molecular Biosystems*, 11(9), pp.2579–2587.

Weickert, M.J. and Adhya, S., 1993. The galactose regulon of Escherichia coli. *Mol. Microbiol*, 10, pp.245–251.
